# Headache and migraine during pregnancy and puerperium: the MIGRA-study

**DOI:** 10.1007/s10194-011-0329-1

**Published:** 2011-03-26

**Authors:** Elisabeth Volden Kvisvik, Lars Jacob Stovner, Grethe Helde, Gunnar Bovim, Mattias Linde

**Affiliations:** 1 Department of Neuroscience, Norwegian University of Science and Technology, Trondheim, Norway; 2 Department of Neurology and Clinical Neurophysiology, Norwegian National Headache Centre, St. Olavs University Hospital, 7006 Trondheim, Norway

**Keywords:** Breast-feeding, Headache, Migraine, Postpartum, Pregnancy, Women

## Abstract

There is little prospectively gathered data on the course of headaches during pregnancy and postpartum, and the influence of breastfeeding is unclear. This is a large, prospective study, which invited all pregnant women in the catchment area during a defined period. All participants (*n* = 2,126) filled in questionnaires concerning headache. Among these, a total of 208 women with migraine according to the International Headache Society criteria also filled in detailed headache diaries during pregnancy and the puerperal period. Freedom from earlier headaches during pregnancy was significantly more common than new onset of headache during pregnancy (*p* < 0.001). This was not influenced by prior use of oral contraceptives. According to the diaries, there was a gradual decrease during pregnancy in the frequency of all headaches and of self-considered migraine. There was also a significant decrease in the duration of headaches (*p* < 0.001) during pregnancy compared to before. Earlier parity did not influence the course. Apart from a significant increase during the first week postpartum (*p* < 0.01), the overall occurrence of headaches during puerperium did not differ from the pregnancy period. Compared to pregnancy, there was a postpartum increase in the mean intensity (*p* < 0.01) and duration (*p* = 0.050) of headaches, as well as in the mean number of analgesics used (*p* < 0.001). Breastfeeding did not influence the occurrence of headaches postpartum. These data are of practical value for informing pregnant migraineurs about the typical clinical prospects and for giving advice on breastfeeding.

## Introduction

Migraine is a disabling disorder that among adults is more prevalent among women than men. This is, at least partly, due to the influence of female sex hormones, as migraine varies with female reproductive events like menarche, the menstruation cycle and the use of oral contraceptives (OCs), pregnancy and menopause [1–3].

Several studies have shown that about one-half to three-fourths of female migraineurs experience a reduction in the frequency or total cessation of migraine attacks during pregnancy [1–12], mainly in the second and third trimesters [5, 7, 11, 13, 14]. In addition, the average pain intensity of the remaining attacks decreases as the pregnancy proceeds [10]. If migraine is not improved by the end of the first trimester, it is likely to continue throughout the pregnancy and postpartum periods [15]. The improvement seems to be especially prominent in women with perimenstrual or pure menstrual migraine [1, 3, 4, 7, 9], and one study has shown that women having migraine without aura are more likely to become better than women having migraine with aura [2].

Inversely, about 8% of pregnant women with migraine experience an increase in the attack frequency and pain intensity of migraine throughout pregnancy [16]. This affects more often those with migraine with aura [5, 9, 17]. New-onset migraine can also occur during pregnancy [3, 5, 12, 15, 17–20]. This concerns about 1.3–16.5% of pregnant women [16], and usually takes place during the first trimester [12, 17]. Migraine occurring for the first time in pregnancy is more likely to be with aura [1].

The majority of data available on the course of migraine during pregnancy are retrospective [1–5, 8, 12, 18, 21]. According to a recent review, this causes a methodological problem, since it appears that the time elapsing between the pregnancy and the interview attenuates the effect of pregnancy thus causing a major bias [22]. By contrast, only a few studies have investigated the course of migraine and other headache during pregnancy prospectively [11, 15, 23], and these suffer from methodological limitations such as small [11, 15] or strongly selected study samples [15, 23], not applying the diagnostic migraine criteria of the International Headache Society (IHS) [23, 24], or a very rough assessment of the course of headache [23].

Postpartum headache is common, occurring in about 30–40% of all women, not only migraineurs [10, 11, 25]. In general, headaches after birth are strongly associated with the presence of a previous or family history of migraine [25, 26]. There is very little scientific data available on the course of migraine and other headache in the puerperium [22]. Most postpartum headaches seem to occur during the first week, and about one half of the women who experience improvement of migraine during pregnancy have recurrence of their ordinary migraine pattern a short time after delivery [10, 25–27].

The influence of breastfeeding on the course of headache and migraine in the puerperium is not yet established. There are few studies on the subject, some of which indicate that breastfeeding impedes migraine from recurring [10, 11]. It is possible that the stable oestrogen levels due to the absence of menstruation during lactation may be protective.

The pregnancy and puerperium pose particular challenges for women with migraine due to the scarcity of treatment options in these periods. The main aims of the present study were to describe prospectively and in detail the overall course of all headaches collectively (regardless of diagnosis) and also of migraine (self-considered migraine among the subgroup of women who fulfil the diagnostic criteria for migraine) during pregnancy and puerperium, and to explore the relation to various factors, in particular breastfeeding. In addition, the influence of factors occurring before the pregnancy, and the effect of becoming pregnant on the headache pattern were explored.

## Methods

### Recruitment, collection of data and diagnostic procedures

The present study is part of the MIGRA-study, in which all pregnant women between May 1997 and June 1998 in the catchment areas of The University Hospital in Trondheim and a nearby hospital (Orkdal Sanitetsforenings Sjukehus) were invited to participate. The invitation accompanied the appointment letters to routine ultrasound in week 18–20 of pregnancy. Those with high-risk pregnancies or known foetal complications were excluded. The original purposes of the MIGRA-study were to examine the occurrence of focal neurological phenomena during pregnancy and to perform a follow up study for 5 years, both of which have previously been published [20, 28].

All participants were asked to respond to three questionnaires. The first (Q1) was sent out together with the appointment letter and handed in during a routine ultrasound. On this occasion, they also received the second questionnaire (Q2) that they were asked to fill in towards the end of the pregnancy. Q2 was collected in when the women were admitted for the delivery. During the stay at the maternity ward, the women received the third questionnaire (Q3) that was returned by mail approximately 8 weeks after birth. These three questionnaires were very similar in design, including questions about headache and its characteristics, focal neurological symptoms, family history and possible risk factors before (Q1) and during (Q2) the pregnancy, and after delivery (Q3). In addition, Q1 included information about former births and spontaneous abortions, number of children, and use of contraceptives before the pregnancy.

The headache questions were mainly designed to determine whether or not the woman had headache and to classify the headache as migraine or not using a modified version of the current migraine criteria of the Headache Classification Committee of the IHS [24]. The diagnostic questions of the questionnaires were identical to those used in the Head-HUNT study, a large population-based survey in the same area performed 2 years earlier. The validity of these questions has been evaluated previously [29]. In uncertain cases, the participants were contacted by telephone to clarify if the headache could be diagnosed as migraine. In women with headache of a non-migraine type, no clear diagnosis was established. Additional questions were included to capture predefined self-perceived transient focal neurological symptoms (numbness/tingling in one arm and/or half face, paresis in one arm and/or one leg, and/or visual disturbances such as flashes, zigzag lines, fog patches or blurred vision).

Participants who fulfilled the criteria for migraine in Q1 were asked to keep a headache diary throughout their pregnancy and during the first 2 months of the puerperium. On a daily basis, they recorded headache intensity (scale 0–4), nausea, vomiting, photo- and phonophobia, attack duration (hours), doses and type of acute medication used, predefined focal neurological symptoms (numbness, paresis, and/or visual disturbances), whether or not they considered the headache to be migraine, and level of functioning (scale 0–3, where 0 is normal and 3 is bedridden).

### Data handling and statistical methods

The diary information was summed up per week. Week −1 was the last week before giving birth and week +1 was the first week after giving birth. The day of delivery was included in week −1. Weeks with missing data on more than 2 of 7 days were coded as ‘missing’ for the relevant week. For the different headache characteristics, the scores were calculated as the sums of the scores for every week divided by 7, giving a mean score for each headache day during a week. Only diaries from week −19 to week +8 are used in the analysis. To investigate more closely the course of all headaches combined and of self-considered migraine around delivery, diary data were plotted for each day from 14 days before to 14 days after giving birth. In the most detailed time course analyses, only the subgroup that had complete diary data from week −19 to week +8 were included, below referred to as the ‘strict migraine diary group’ (*n* = 66). To explore the possible bias created by the exclusion of participants with incomplete diaries, analyses of all participants with available calendars, below referred to as the ‘liberal migraine diary group’ (*n* = 208) and the ‘strict migraine diary group’ were compared. Due to the small samples, the complete ‘liberal diary group’ was included in the comparison of calendars from breastfeeders versus non-breastfeeders.

For statistical analysis of paired data (before vs. during pregnancy, and before vs. after delivery) McNemar’s test was used on categorical, dichotomous variables (diagnoses), and Wilcoxon Signed Rank test on variables on an interval level (categories of attack frequency and duration in questionnaires), since normal distribution could not be assumed. Paired samples *t* test was used to compare continuous variables (e.g. diary data on mean number of attacks in different periods). To compare independent groups (e.g. those with improvement versus those with deterioration during pregnancy, nulli- versus multipara, breast-feeders versus non-breast-feeders), independent samples *t* tests and Chi-square tests were performed on continuous and categorical variables, respectively. A *p* value <0.05 was considered statistically significant. The statistical analyses were performed using the Statistical Package for the Social Sciences (SPSS), version 15.0.

## Results

### Participation

**Fig. 1:**
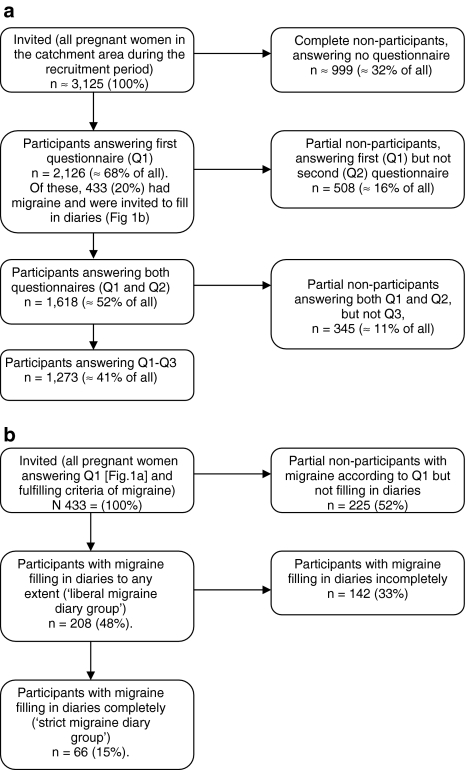
**a** Flow of participants through the different steps of the questionnaire survey **b** Flow of participants through the different steps of the diary survey

The flow of participants through the different steps of the survey is shown in Fig. 1a, b. Among all invited (approximately between 3,000 and 3,250), 2,126 (mean age 28.4 years) answered Q1. Among these, 1,618 (76%) answered Q2, and 1,273 (60%) participated in Q1–Q3. A total of 433 (20%) women had migraine according to Q1 and were invited to fill in the diary during pregnancy and the puerperal period. Among them, 208 (48%) filled in calendars to at least some extent (‘liberal migraine diary group’). At the most, some participants kept the diary from week −27 to week +9, but the majority kept it for a shorter period. Among the ‘liberal migraine diary group’, 206 (99%) answered Q2, and 158 (76%) participated in Q1–Q3.

Major demographic characteristics of participants and non-participants are shown in Table 1. The mean age in the ‘liberal migraine diary group’ was 29.5 years (median 29, range 17–44).

**Table 1:** Demographic characteristics within the different groups

	Mean age years ± SD	Primipara *n* (%)	Smoking before pregnancy *n* (%)	Elevated blood pressure before/during pregnancy *n* (%)	Other complaints during pregnancy *n* (%)
Participants Q1 (*n* = 2,126)	28.4 ± 4.8	893 (42)	936 (44)	Before: 47 (2)	–
Partial non-participants (answered Q1 only) (*n* = 508)	27.7 ± 5.1	213 (42)	252 (50)	Before: 12 (2)	–
Partial non-participants (answered Q1 + Q2 only) (*n* = 345)	28.5 ± 4.7	153 (44)	160 (46)	Before: 6 (7)	169 (49)
‘Liberal migraine diary group’ (*n* = 208)	29.5 ± 4.6	83 (40)	73 (35)	Before: 73 (4)	107 (51)
				During: 23 (11)	
Partial non-participants (diagnosed migraineurs not filling in calendar) (*n* = 225)	28.3 ± 4.9	76 (34)	112 (50)	Before: 9 (4)	62 (28)
				During: 20 (9)	
‘Strict migraine diary group’ (*n* = 66)	29.5 ± 4.7	28 (42)	15 (23)	Before: 2 (3)	35 (53)
				During: 9 (14)	
Partial non-participants not filling in diaries completely (*n* = 142)	29.4 ± 4.8	55 (39)	58 (41)	Before: 5 (4)	72 (51)
				During: 14 (10)	

‘Other complaints during pregnancy’ were mainly pelvic pain, nausea unrelated to headache, back pain and oedema

### New onset of having headache versus freedom from any earlier headache during pregnancy

More women changed their answer from having any headache before pregnancy (Q1) to not having any headache during pregnancy (Q2) (248/1,618 = 15%) than the other way around (101/1,618 = 6%) (*p* < 0.001). The group with onset of headache during pregnancy (*n* = 101) and the group with freedom from earlier headache during pregnancy (*n* = 248) did not differ significantly with regard to age (mean 28.0 and 28.4 years, *p* > 0.2) or earlier parity (0.77 and 0.78 children, *p* > 0.2). Nor did the use of OCs (*p* > 0.2) or the presence of high blood pressure prior to pregnancy differ between the two groups (*p* > 0.2).

### Change in frequency of all headaches combined during pregnancy and puerperium

According to categorical data in questionnaires there was no significant difference (*p* = 0.12) in the frequency of all headaches combined during pregnancy (Q2, *n* = 1,122 had headache and answered) compared to before (Q1, *n* = 1,269 had headache and answered) among women who participated in both questionnaires (*n* = 1,618).

**Fig. 2:**
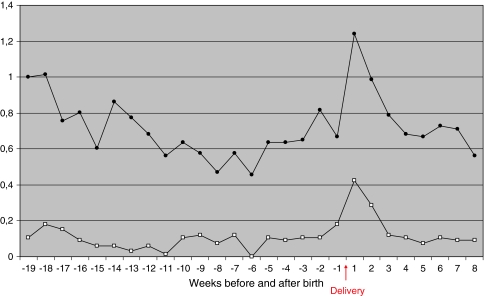
Variation of the mean frequency of all headaches combined (*upper graph*) and self-considered migraine (*lower graph*) during pregnancy (second half) and puerperium among migraineurs with complete diaries (*n* = 66)

Based on the diary data from the ‘strict migraine diary group’, the course of all headaches combined was analyzed in detail during the second half of pregnancy and puerperium (Fig. 2). The initial mean frequency was 1.0 days/week, decreasing to 0.6 days/week from about week −12. There was a marked increase of episodes during the first week after giving birth. There was no significant change in the mean frequency before and after birth, but there were significantly more headache days during the first week after giving birth than the average for the pregnancy period (*p* < 0.01).

**Fig. 3:**
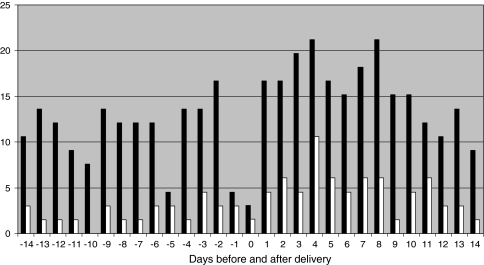
Variation in point-prevalence (daily %) of all headaches combined (*black bars*) and self-considered migraine (*white bars*) during the period from 2 weeks before delivery until 2 weeks after delivery among migraineurs with complete diaries (*n* = 66)

Figure 3 shows the proportion of women in the ‘strict migraine diary group’ reporting any headache day by day from week −2 to week +2. On the day of delivery, only two of the women reported headache.

Among women in the ‘liberal migraine diary group’, the mean number of children was 1.0 (median 1, range 0–5). No significant differences (data not shown) between nulli- (*n* = 83) or multiparous (*n* = 125) women were found with regard to frequency of overall headache before the current pregnancy (according to retrospective question in Q1), during the pregnancy (Q2) or in the first 8 weeks of the puerperium (Q3).

### Change in frequency of self-considered migraine during pregnancy and puerperium among migraineurs

In the ‘strict migraine diary group’, the relative course of self-considered migraine (Fig. 2) was very similar (on a lower level) to that of all headaches combined. The mean frequency of self-considered migraine varied around 0.1 days/week during pregnancy until delivery. The mean frequency of self-considered migraine was significantly higher (*p* < 0.01) for the postpartum period (0.16 days/week) than for the pregnancy period (0.09 days/week), mostly due to the peak around delivery. Compared to the entire pregnancy period, there was a significant increase of self-considered migraine during week +1 (*p* = 0.013) and week +2 (*p* = 0.028).

As seen in Fig. 3, only one woman of the 66 migraineurs with complete diaries had self-considered migraine on the day of delivery.

### Characteristics of headaches during pregnancy and puerperium among migraineurs

There was a significant shortening of duration of headache episodes (*p* < 0.001, categorical data) during pregnancy (Q2) compared to before (Q1) among all participating women.

Comparing pregnancy and puerperium, there was a postpartum increase in the mean headache intensity (*p* < 0.01), the mean headache duration (*p* = 0.050), and the mean number of analgesics used (*p* < 0.001) among all women in the ‘liberal migraine diary group’. No significant differences were found for presence of nausea, vomiting, photo-/phonophobia, or functioning level during attacks.

### Role of breastfeeding on the course of headaches during puerperium

**Fig. 4:**
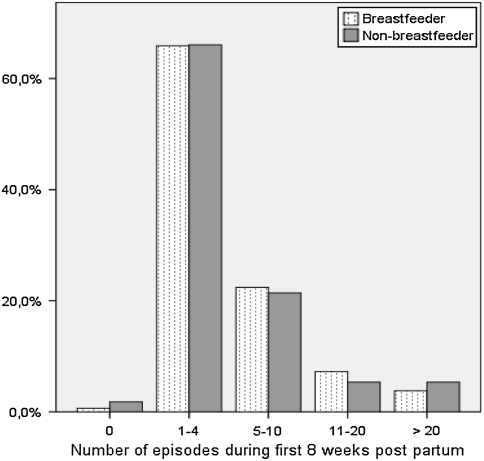
Distribution of breastfeeding (*n* = 607) and non-breastfeeding (*n* = 56) women among different categories of frequency of any headache (number of attacks during the first 8 weeks) of the puerperium

The relation between breastfeeding and the experience of any headache during 8 weeks postpartum was first analysed in all women answering this question in Q3 (*n* = 1,271). There was no significant difference (*p* > 0.3) between the proportion with puerperal headache among breastfeeders (621/1,175, 53%) and non-breastfeeders (56/96, 58%). The distribution of breastfeeders and non-breastfeeders on different categories (Q3) of headache frequency during puerperium is presented in Fig. 4. The majority of both breastfeeders and non-breastfeeders had 1–4 headache attacks in the 8-week period.

**Fig. 5:**
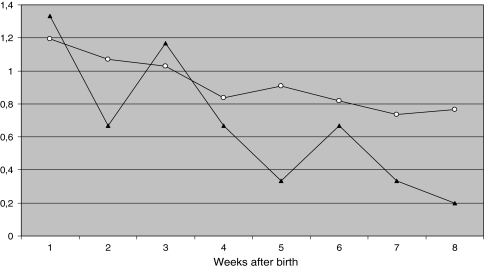
Course of overall headache (calendars) during the puerperium for breastfeeding (*white circles*, *n* = 149) and non-breastfeeding (*black triangles*, *n* = 9) migraineurs

In the ‘liberal migraine diary group’, 158 answered the question in Q3 (*n* = 158) on whether they had breastfed their newborns (*n* = 149) or not (*n* = 9) during the first 8 weeks postpartum. Among them, there were no significant differences in mean overall headache days per week (Fig. 5) for breastfeeders compared to non-breastfeeders, neither for each individual week (all *p* > 0.1) nor for the whole period (*p* > 0.1).

**Fig. 6:**
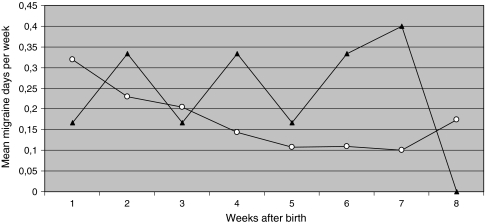
Course of self-considered migraine (calendars) during the puerperium for breastfeeding (*white circles*, *n* = 149) and non-breastfeeding (*black triangles*, *n* = 9) migraineurs

In the ‘liberal migraine diary group’, breastfeeders had significantly fewer days with self-considered migraine than non-breastfeeders in week +7 (*p* < 0.001), but for the whole postpartum period there was no significant difference in the occurrence of self-considered migraine between breastfeeders and non-breastfeeders (Fig. 6).

**Fig. 7:**
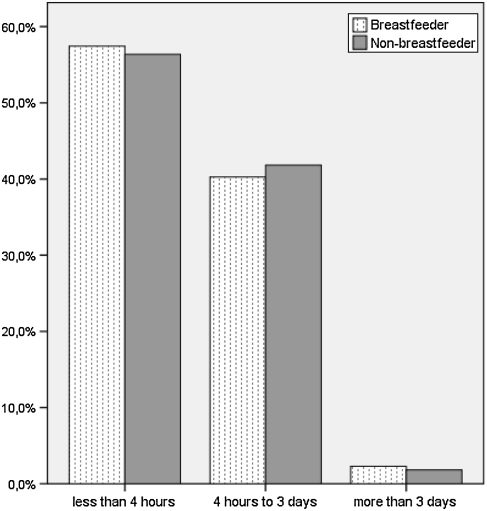
Distribution of breastfeeding (*n* = 611) and non-breastfeeding (*n* = 55) women among different categories (questionnaire data) of duration of headaches in general during the puerperium

Among all participants, the duration of episodes of headache in general were reported to be very similar in breastfeeders and non-breastfeeders, the majority in both groups having headaches lasting <4 h (Fig. 7).

In the diaries from the ‘liberal migraine diary group’, the mean intensity (scale 0–4) of headache in general was reported as 1.9 among the breastfeeders (*n* = 149) and 2.2 among the non-breastfeeders (*n* = 9) (*p* = 0.12). The mean headache duration was 9.0 h and 8.7, respectively (*p* > 0.2).

## Discussion

### Methodological considerations

Major strengths of the present study are that all pregnant women in a certain period were invited prospectively, thereby avoiding selection bias, and that the number of women answering the questionnaires (from *n* = 2,126 for Q1 to *n* = 1,273 for Q3) and migraineurs volunteering to fill in calendars (*n* = 208) are the largest published so far for this type of study. Other strong points of the methodology are the detailed questionnaires and diaries, and that the IHS-criteria were used for diagnosing women with migraine. The study by Sances et al. [10] was done more than 20 years before the IHS-criteria were introduced. They included 47 patients with migraine without aura (two with migraine with aura were excluded) in the analyses. Of these, ten (21%) were bottle feeding. In the study by Scharff et al. [11], 30 pregnant women having headache ≥2 times per month were recruited through advertisements in local newspapers and posters at the hospital. The IHS-criteria were used to categorise women into having migraine, tension-type headache, or both.

A methodological limitation of the present study is that two-thirds (*n* = 142, 68%) of participating migraineurs had incompletely filled in calendars. A common reason for exclusion of diaries was that participants had only filled in headache intensity (scale 0–4) on days with headache, so that days with no headache became ‘missing’ instead of ‘0’. In some analyses, only participants with complete data sets (*n* = 66, ‘strict migraine diary group’) were included. Due to the risk of creating a type-II error, the ‘liberal migraine diary group’ was used in the analysis of the role of breastfeeding. During the statistical work-up, the ‘liberal migraine diary group’ and the ‘strict migraine diary group’ were compared to control for bias. Although there were some differences (reported below), the main trends were similar in both groups.

Another weakness of the study is that each headache episode in calendars was not diagnosed as migraine or non-migrainous headache based on the features reported in the calendar before being entered into the computer. Rather, self-assessment of having migraine on days with headache was used. The fact that self-considered migraine constituted only a small proportion of all headaches among the migraineurs points to low awareness of the definition of migraine among the participants, indicating that the result should be interpreted with caution. However, since the curves were very similar for headache and migraine, we believe that the results are fairly representative for migraine during pregnancy and puerperium.

### New onset of having headache versus freedom from any earlier headache during pregnancy

The finding that many women with earlier headache became headache free during pregnancy is consistent with several other studies [1–12]. It is interesting to note that this favourable course was more than twice as common as developing new-onset headache. None of the studied variables can be used as prognostic factor.

### Change in frequency of all headaches combined and of self-considered migraine among migraineurs during pregnancy and puerperium

Our data are in concord with previous studies [10, 11, 15] showing a gradual decrease during pregnancy of headaches in general and self-considered migraine towards birth.

Scharff et al. [11] reported an increase in headache activity around birth and the following 3 weeks, thereby supporting our finding. The peak following delivery may be a result of several factors such as the physical strain of giving birth and the ensuing sleep deprivation and adaptation to the new postpartum situation, as well as psychological factors like anxiety and worry. In addition, during labour several hormones are interacting and possibly affecting neurotransmitter systems in the brain which may produce headache. One should also consider the possibility that headaches in the early postpartum period may occur as side effects of procedures or medication during labour, for example spinal puncture headaches after epidural or spinal anaesthesia or use of NO_2_-gas.

It is interesting that the overall occurrence of headache among migraineurs postpartum is as low as in pregnancy in spite of the marked peak in incidence right after delivery. This may have several possible explanations. One is that breastfeeding women lack menstruation and, therefore, have relatively stable hormonal levels. The women may also have adapted to increased pain after giving birth, physiologically and perhaps psychologically. In addition, we found that many women start using analgesics in the puerperium, possibly for other conditions than headache, and this may also prevent headaches.

The present study showed no significant effect of parity on the course of headaches during pregnancy among migraineurs. This confirms the finding in an earlier study where headache activity in pregnancy was not influenced by parity [15]. Furthermore, both nulli – and multipara migraineurs showed a sustained improvement of headache also during the puerperium. In contrast, Scharff et al. [11] found U-shaped curve for headache during pregnancy in multiparous women with an increase at the end of the third trimester. Also, a study by Stein et al. [25] reported that headaches after birth were more frequent among multipara, and that a larger proportion of the women in this group had a migraine history.

In the ‘strict migraine diary group’, there were significantly more self-considered migraine days postpartum than during pregnancy. The small mean increase (0.07 days/week) is not likely to be of clinical significance. In the ‘liberal migraine diary group’, the frequency of self-considered migraine during puerperium was not significantly different from the pregnancy period.

### Characteristics of headaches during pregnancy and puerperium among migraineurs

The intensity and duration of headache were significantly reduced in pregnancy among the ‘liberal migraine diary group’, and the differences were even more pronounced in the ‘strict migraine diary group’ (data not shown).

The number of doses of acute medication was lower in pregnancy, which probably reflects a general cautiousness in Norway with using medication during pregnancy. The finding is consistent with that of Maggioni et al. [5] who reported that use of medication during pregnancy was reduced and restricted to a limited number of compounds.

### Role of breastfeeding on the course of headaches during puerperium

There were no significant differences between breastfeeding and non-breastfeeding women regarding the occurrence of headaches postpartum. As to the effect of breastfeeding on the activity of headache in general, the data have been relatively scarce. Sances et al. [10] found that breastfeeding was associated with delayed recurrence of migraine postpartum. In the study of Scharff et al. [11], there were too few women who were not breastfeeding to analyse differences in the headache postpartum, but the authors nevertheless suggested that the fact that headache activity postpartum was at the same low level as in pregnancy could be due to the positive influence of breastfeeding. However, the current study could not confirm this, neither with regard to the frequency, intensity or duration of headache. Admittedly the influence of breastfeeding upon headache was difficult to assess using the diaries because very few were not breastfeeding. The findings of the current study are in accordance with Wall’s case series which showed no significant effects of lactation on headache activity in a large group of lactating migraineurs [30]. Likewise, another study reported no significant association between breastfeeding and headache improvement from the second trimester to the postpartum period [15].

## Conclusion

We believe our results may be of considerable practical value in informing the pregnant migraine sufferer about the prospects in pregnancy and the postpartum period. Also, our data may be of theoretical interest for understanding the influence of sex hormones on headache in general and particularly among migraineurs.

## Abbreviation

IHS: International Headache Society
